# Single-Photon Detectors on Arbitrary Photonic Substrates

**DOI:** 10.1021/acsphotonics.5c00345

**Published:** 2025-04-22

**Authors:** Max Tao, Hugo Larocque, Samuel Gyger, Marco Colangelo, Owen Medeiros, Ian Christen, Hamed Sattari, Gregory Choong, Yves Petremand, Ivan Prieto, Yang Yu, Stephan Steinhauer, Gerald L. Leake, Daniel J. Coleman, Amir H. Ghadimi, Michael L. Fanto, Val Zwiller, Dirk Englund, Carlos Errando-Herranz

**Affiliations:** † Research Laboratory of Electronics, 2167 Massachusetts Institute of Technology, Cambridge, Massachusetts 02139, United States; ‡ 7655 KTH Royal Institute of Technology, 106 91 Stockholm, Sweden; § 122364 Centre Suisse d’Electronique et de Microtechnique (CSEM), 2000 Neuchâtel, Switzerland; ∥ Raith America Inc., Troy, New York 12180, United States; ⊥ State University of New York Polytechnic Institute, Albany, New York 12203, United States; # Air Force Research Laboratory, 97040 Information Directorate, Rome, New York 13441, United States; ○ Institute of Physics, University of Münster, Münster 48149, Germany; □ QuTech and Kavli Institute, 2860 Delft University of Technology, 2628 Delft, The Netherlands; △ Department of Quantum and Computer Engineering, Delft University of Technology, 2628 Delft, The Netherlands

**Keywords:** quantum photonics, single-photon detectors, optical quantum technologies, photonic integrated circuits, superconducting nanowire single-photon detectors

## Abstract

Detecting nonclassical light is a central requirement for photonics-based quantum technologies. Unrivaled high efficiencies and low dark counts have positioned superconducting nanowire single-photon detectors (SNSPDs) as the leading detector technology for integrated photonic applications. However, a central challenge lies in their integration within photonic integrated circuits, regardless of material platform or surface topography. Here, we introduce a method based on transfer printing that overcomes these constraints and allows for the integration of SNSPDs onto arbitrary photonic substrates. With a kinetically controlled elastomer stamp, we transfer suspended SNSPDs onto commercially manufactured silicon and lithium niobate on insulator integrated photonic circuits. Focused ion beam metal deposition then wires the detectors to the circuits, thereby allowing us to monitor photon counts with >7% detection efficiencies. Our method eliminates detector integration bottlenecks and provides new venues for versatile, accessible, and scalable quantum information processors.

## Graphical abstract

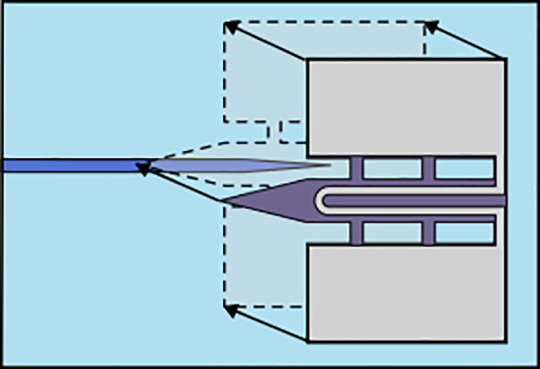

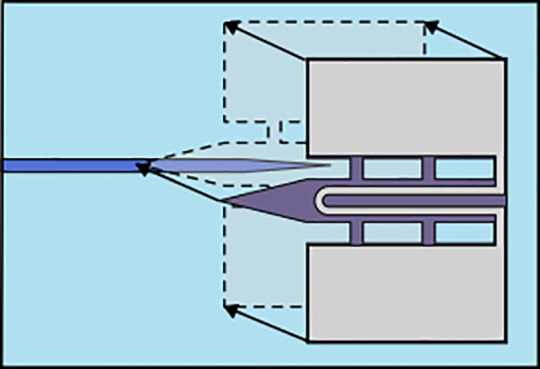

## Introduction

Optical quantum technologies are central to quantum computing,^1^ communication,^2^ and simulation.^3^ Scaling these technologies to the system sizes required by quantum applications has motivated the development of quantum photonic integrated circuits (PICs),^4^ which leverage standardized semiconductor manufacturing for producing large-scale optical systems. A key requirement of such systems is the detection of single photons for tasks ranging from preparing and measuring quantum states to implementing quantum gates.^5^ Superconducting nanowire single-photon detectors (SNSPDs) are among the best single-photon detectors available today due to their combination of record high detection efficiency,^6^ broadband operation,^7^ low dark counts,^8^ fast recovery time,^9^ very low timing uncertainty,^10^ and compatibility with photonic reconfiguration.^11^

A central challenge in the development of quantum PICs involves integrating SNSPDs within large-scale circuits with (i) sufficient fabrication yields and (ii) universal methods that seamlessly carry over within PIC platforms. Monolithic integration can readily provide sufficient yield levels, yet often involves process flows hyper-specialized to a particular fabrication node.^12,13^ To address this issue, recent advances in hybrid quantum PICs^14,15^ motivated the development of micrometer-scale flip chip processes for integrating SNSPDs on a wider range of PICs.^16^ However, successful flip chip transfers require meticulous handling of the SNSPDs with equipment, such as tungsten microprobes. Furthermore, this method requires highly accurate structural features conforming with those of SNSPDs, not present in the vast majority of PIC platforms. Both of these drawbacks could prevent deploying SNSPD flip-chip transfers at scale, thereby warranting a hybrid integration method that simultaneously overcomes: (i) fabrication incompatibilities among PIC platforms, with a prominent example being lithium niobate on insulator (LNOI) which require customized SNSPD fabrication flows,^13,17–19^ (ii) limited device yields,^7,12^ and (iii) lack of control over the PIC fabrication process, which can be especially common while integrating with large-scale foundry-processed PICs.^20,21^

Here, we address these challenges via the hybrid integration of SNSPDs on PICs via transfer printing. Our method relies on (i) standardized SNSPD fabrication^11^ to avoid incompatibilities with PIC fabrication, (ii) preliminary screening allowing us to selectively transfer functioning devices and overcome yield limitations in monolithic PIC platforms, and (iii) structures compatible with arbitrary PICs that do not require flip chip bonding. We demonstrate the versatility of our method by integrating SNSPDs onto large-scale foundry silicon PICs and LNOI PICs, thereby confirming its compatibility with PIC platforms ranging from commercially manufactured systems to those that otherwise require substantially tailored SNSPD integration processes.

## Detector Fabrication

Our device fabrication consists of (i) fabricating suspended silicon nitride waveguides topped with hairpin SNSPDs, (ii) detector screening via room-temperature resistance measurements, (iii) transfer printing, and (iv) wiring to ensure electrical connectivity. The fabrication of our detectors draws on a process developed for MEMS-actuated PICs with SNSPDs.^11^ As outlined in Supporting Section 1, the process produces suspended silicon nitride photonic waveguides topped with a NbTiN hairpin detector. Prior to transfer printing these devices, we measured their resistance at room temperature for screening purposes and selected devices exhibiting a finite resistance between 1 and 2 MΩ for transfer. Supporting Section 1 provides statistics on the measured resistances.

Drawing on established integration methods for on-chip light sources,^22,23^ we rely on a kinetically controlled elastomer stamp to transfer these SNSPDs on PICs. An optical microscopy apparatus loaded with a 0.41 numerical aperture objective allows us to monitor this transfer to preserve a sufficient level of alignment between the PIC and the detectors. Figure 1a provides schematics of this integration process. The resulting devices feature hybrid optical mode converters formed by the PIC’s native and the SNSPD’s nitride waveguides, thereby enabling optical connectivity between the PIC and the transferred devices. To ensure electrical read-out from the SNSPD, we connect it to the PIC’s electrical lines. This is done via in situ focused-ion-beam (FIB) chemical vapor deposition of tungsten wires (see Figure 1b and Supporting Section 2 for more information).

**Figure 1:**
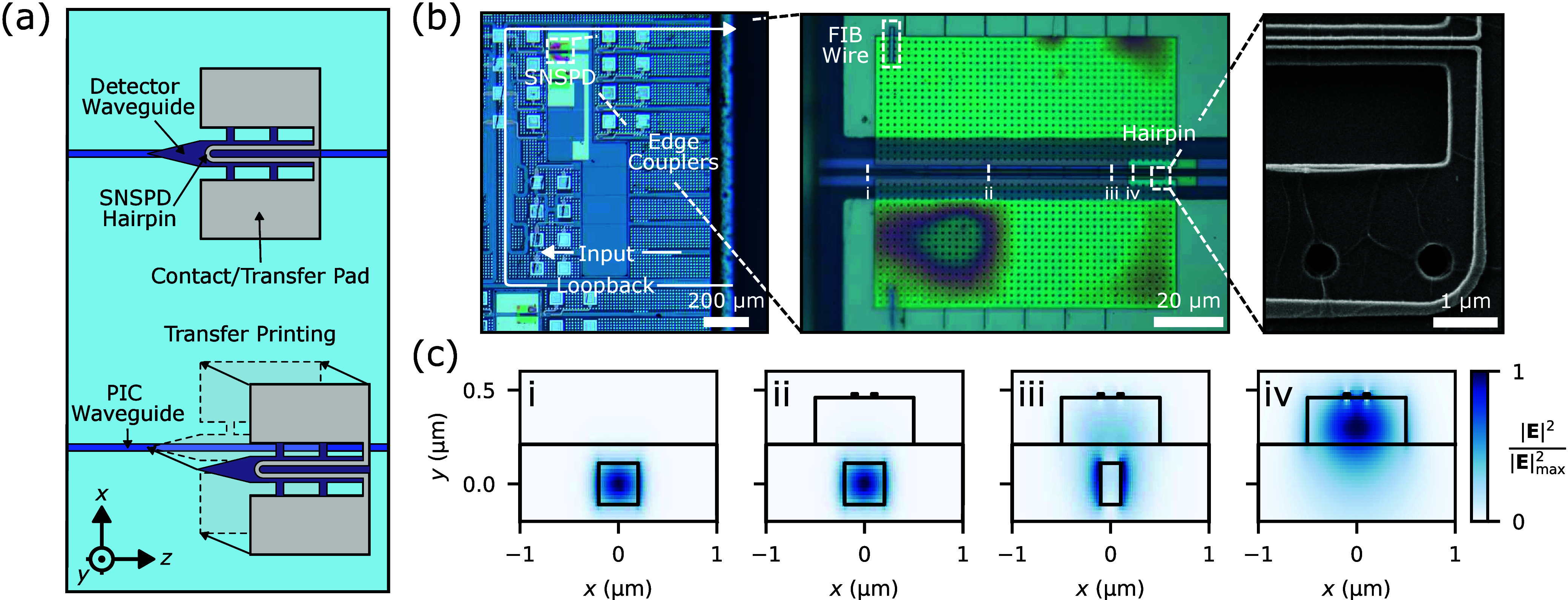
Hybrid integrated SNSPD assembly and modeling. (a) Schematics of the hybrid integration process of SNSPDs on foundry PICs. (b) Optical micrograph of a foundry silicon PIC with integrated SNSPDs. Insets: optical micrograph of an assembled device, and a scanning electron micrograph of the hairpin detector. (c) Fundamental mode profile of the hybrid mode converter at distances of (i) 0, (ii) 56, (iii) 96, and (iv) 100 μm from the start of the structure’s detector waveguide. These positions respectively correspond to the start of the detector waveguide, the start of the taper in the PIC waveguide, the end of the taper in the PIC waveguide, and a region where the mode converter uniquely comprises of the detector waveguide.

We then tested the hybrid SNSPD-PIC structures in a closed loop cryostat with a base temperature of 0.78 K. Further details on the experimental setup are available in Supporting Section 3. We confirm our detectors’ ability to monitor photon counts by flood illuminating our chip before measuring their on-chip detection efficiency (ODE).

## Integration on Foundry Silicon Photonics

We first integrate our SNSPDs on PICs commercially manufactured using a 193 nm deep-ultraviolet water-immersion lithography silicon photonic process.^20^ Figure 1b provides optical and scanning electron micrographs of the assembled structure. We design the resulting hybrid device for detection of the PIC waveguide’s TE0 mode and adopt a mode converter to route this mode into the SNSPD. Figure 1c shows the resulting hybrid TE mode’s profile at various points across the converter. As further elaborated in Supporting Section 4, the PIC consists of single mode silicon waveguides operating at the O and C+L bands. FEM simulations suggest an optical absorption of 30.3% at 1570 nm wavelengths, i.e., given the adopted device geometry shown in Figures 1b,c, we expect a maximum ODE of 30.3% even if most of the light in the PIC waveguide’s TE0 mode transfers into the detector waveguide at the end of the tapered PIC waveguide. This figure also incorporates the loss arising from a 92.5% TE0 mode transmission efficiency at the end of the PIC taper. However, we expect an observed angular offset between the PIC and detector waveguides to reduce our detection efficiency to 8.7% .

We cryogenically test the hybrid SNSPD-silicon PIC device to confirm superconducting behavior, measuring a switching current of 7.1 μA (see Supporting Section 5 for the *I*–*V* curves). We measure their ODE at C+L- and O-band telecommunication wavelengths compatible with the silicon waveguides of our PIC. We send 1570 and 1312 nm light from tunable external cavity diode lasers through a variable optical attenuator followed by a UHNA1 optical fiber array before going in the PIC by means of edge couplers at the chip’s facet. We measure the optical transmission through the various stages of this fiber line (see Supporting Table 2 for these precharacterized values). We then confirm that the device can monitor counts by measuring a characteristic output pulse from the SNSPD under illumination through the PIC (see Supporting Figure 11­(b)).

Figure 2 plots the resulting photon and dark counts monitored by the detector, showing clear plateaus at both wavelengths that indicate a high internal detection efficiency with low dark counts. We attribute the discrete and atypical appearance of our dark count data to the 100 ms integration time of our apparatus. This short integration setting also makes systematic fluctuations in our measurement apparatus more noticeable, which otherwise become negligible while recording photon counts. While biasing the detector with currents of 6.6 μA/6.2 μA, we measured photon count rates of 1.300 MHz/509 kHz for input wavelengths of 1570 nm/1312 nm. As shown in Supporting Section 5, we confirm that these count-rates linearly drop with the intensity of our input light. The detector features a dark count rate of 40 Hz. We compute the ODE by taking the ratio between the detector’s photon counts and the photon flux, Φ, in the silicon waveguide. Based on our apparatus, we define this metric as

1 $$\Phi =\frac{1}{h\nu }\left[P_{in}\cdot 10^{-\left({dB}_{f}+{dB}_{c}+{dB}_{attn}\right)/10}\right]$$

where dB_f_ is the measured loss in dB through the fiber fed into the cryostat, dB_c_ is the measured loss through the edge coupler, and dB_attn_ is the setting of the variable attenuator. *P*_in_ is the power supplied by the laser and *hν* is the input photon energy. As discussed in Supporting Section 6, we measure dB_c_ with an integrated loopback waveguide near the one coupled to the examined SNSPD. The extracted coupling efficiency value assumes identical optical coupling among the considered fiber-edge coupler pairs and also perfect alignment between the fiber array and the PIC. Based on this estimate, our device showed a waveguide-coupled ODE of 7.6 ± 0.2% at 1570 nm and 7.0 ± 0.1% at 1312 nm at bias currents of 6.6 and 6.2 μA, respectively (see Supporting Section 7 for error calculations). These values share the same order of magnitude as our numerical estimates.

**Figure 2:**
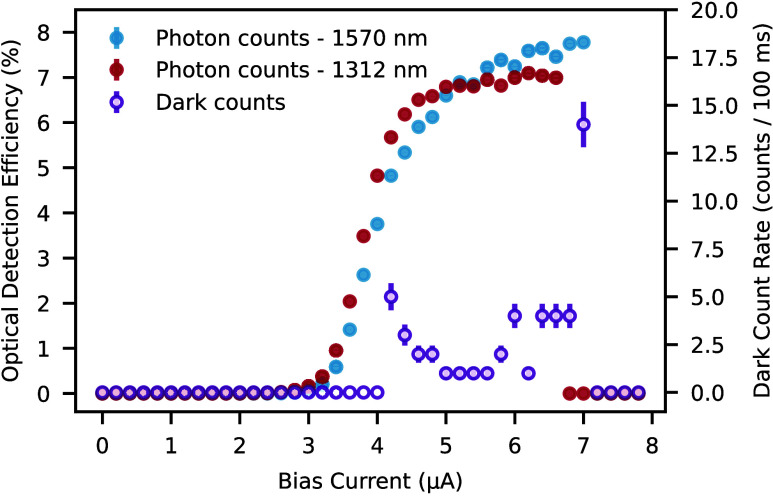
Detection efficiency. On-chip count rates acquired over 100 ms counting times of hybrid integrated SNSPDs for photons propagating through the waveguides of a silicon PIC.

## Integration on Lithium Niobate Photonics

We next illustrate the compatibility of our method with LNOI photonics. Direct SNSPD fabrication on such devices has proven challenging given the need for customized superconductor thin film deposition compatible with lithium niobate that avoids excessive substrate heating.^13,17–19^ Furthermore, adequate precautions must protect photonic waveguides during the detector fabrication^13,19^ or alternatively the detectors during the waveguide fabrication.^18^ Our approach overcomes these issues by fabricating the detectors and waveguides on separate substrates, thereby motivating its integration into other PIC platforms facing similar fabrication challenges. Figure 3a shows the transferred SNSPDs on the LNOI PICs. The LN waveguides consist of 200 nm thick straight waveguides surrounded by a 100 nm ridge. They have a width of 150 nm and a sidewall angle of 55°. We characterize these devices at optical wavelengths of 650 nm with the same methodology used for the silicon PICs, thereby demonstrating low dark counts at saturation and efficiencies of 0.30 ± 0.03%, 4.2 ± 0.2%, and 9 ± 2% for detectors D1, D2, and D3, as shown in Figure 3b, respectively. We attribute these differing values to factors that are absent in our silicon PIC. These factors include fabrication process instabilities leading to unaccounted variations in edge coupler or splitter loss, the excitation of higher order modes in the detector waveguide due to misalignment, and differing parasitic counts due to stray light, as expected from the varying yet close proximity of the edge couplers to the detectors. As mentioned in Supporting Section 7, the lithium niobate chip’s current configuration prevents us from directly isolating the contributions of these effects, which likely justifies why the variation in the three reported efficiencies exceeds their uncertainty. As indicated in the Supporting Section 5, we measure a detector jitter of 242 ps. We then monitored the counts from the three on-chip detectors to gauge the relative influence between waveguide-coupled and stray photons on detected counts. In Figure 3b, we provide the corresponding photon and dark counts while sending light in the edge coupler leading to detector D2. To highlight the different efficiencies of each detector, we normalized the counts to the measured detector efficiencies. Figure 3c plots these counts at saturation, while normalized to the highest counts monitored on detector D2.

**Figure 3:**
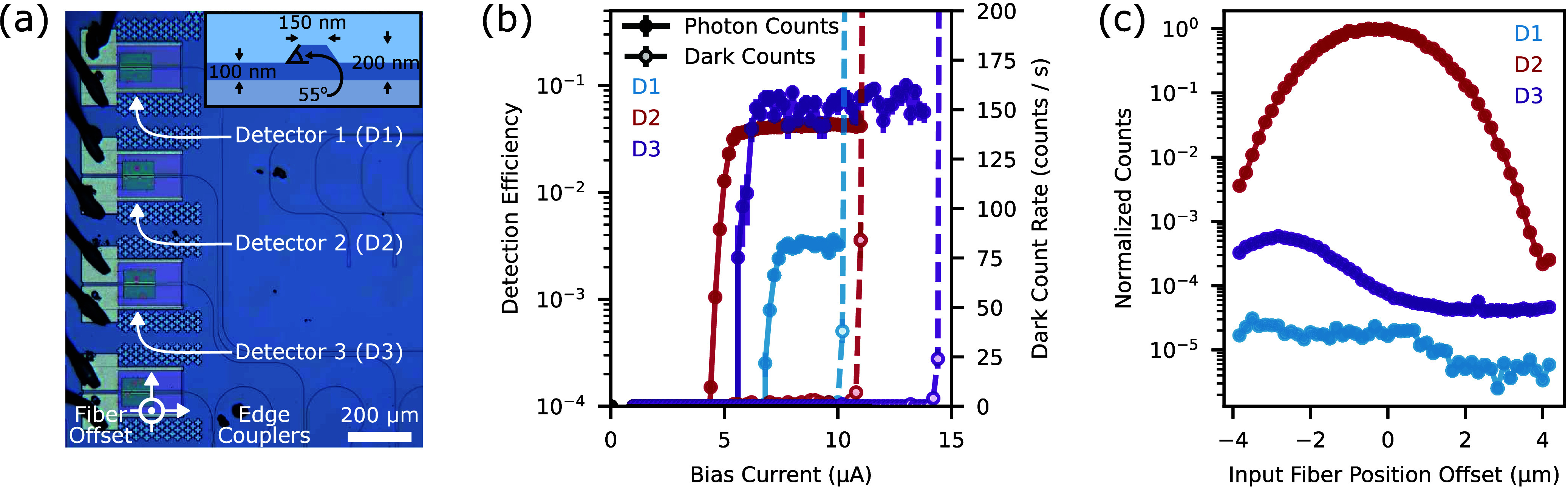
Integration of SNSPDs on LNOI. (a) Optical micrograph of a lithium niobate PIC with three integrated SNSPDs. Inset: schematics of the PIC’s LNOI waveguides. (b) On-chip count rates for the three detectors shown in (a). The reported data is for the detection of light with a 650 nm wavelength while sending light in the waveguide leading to detector D2. (c) Relative count rates of the three detectors vs the relative displacement of the optical fiber sending light into the PIC. The data considers values relative to the position yielding optimal coupling into detector D2. The considered displacement lies along the out-of-plane axis of the micrograph shown in (a).

Under optimal fiber to PIC coupling conditions, we observe a 40 dB extinction ratio between the coupled, D2, and uncoupled, D1 and D3, SNSPDs, or equivalently, between the device’s waveguide-coupled and stray photons. When the fiber is below the waveguiding layer, i.e., for negative fiber position offsets, scattered light propagating through the buried oxide layer toward the detectors leads to 10 dB higher relative counts for D1 and D3.

## Discussion

As outlined in Supporting Section 4, various approaches to codesigning the PIC and detector waveguides can increase detection efficiencies above our 7.6% metric. For instance, extending the length of the detector waveguide can increase the SNSPD’s ODE to values near 99%. Furthermore, adopting measures such as distinct superconducting materials, better waveguide coupling, and reductions in kinetic inductance can lead to detectors with low jitter,^24^ compatibility with longer optical wavelengths,^7^ and shorter reset times.^9^ The angular alignment accuracy also affects mode conversion between the adiabatically tapered substrate and SNSPD waveguides (see Supporting Section 4). Prior hybrid integration work of quantum photonic components on foundry PICs suggest that average offsets of 0.59° are statistically achievable under optimized transfer conditions.^23^ From simulations, we expect detectors with this offset to feature a similar ODE to that of a perfectly aligned device. Alternatively, relying on mode converter geometries that are more tolerant to misalignment could provide a path toward increasing the optical power transferred to the hybrid SNSPD.^25^

As in the case of prior hybrid approaches to SNSPD integration, our approach allows for screening faulty devices to overcome device yield limitations. In addition, our use of elastomer stamps and detector-to-chip wiring gives us access to the full scaling potential of transfer-printing based technologies,^22,23^ which, in industrial settings, can reach transfer rates of up to two billion devices per hour with microscope-limited alignment tolerances.^26^ Though stamp-based printing provides a means for a high-rate and automated detector transfer, its integration still requires the deposition of electrical contact lines. Our tungsten FIB-CVD deposition method conveniently wires the chip to the detectors without damaging them, yet it can be time-intensive. Alternative methods of electrically interfacing detectors, such as optical lithography followed by metal deposition or high precision printing of silver nanoinks can reliably produce our required low resistivity and micron-scale contacts.^27^

In summary, we demonstrated a hybrid integration method for the interfacing of SNSPDs with arbitrary photonic substrates. As surveyed in Supporting Section 8, our method allows for SNSPD integration on PICs regardless of its material platform, chip size, and surface topography. Using transfer printing and FIB-CVD on silicon PICs, we attain detector efficiencies of 7.6% in the C+L band and 7.0% in the O-band along with dark counts of less than 100 Hz. In addition, we transferred devices onto LNOI chips and observed efficiencies of 0.3%, 4.2%, and 8.6%, thereby demonstrating the versatility of our technique in regard to the PIC’s material platform. Our results underscore SNSPD integration onto arbitrary PICs ranging from those manufactured at scale in a commercial foundry^20,21,23^ to those where monolithic detector fabrication can compromise the integrity of the PIC.^13,17–19^ Enabling accessible integration of state-of-the-art single-photon detectors onto scalably manufacturable PICs implemented in arbitrary material platforms opens the door to fully integrated quantum technologies for applications ranging from quantum communications with quantum repeaters^28^ to measurement-based quantum computing,^29^ quantum sensing,^30^ and computing with trapped ions.^31^

## Supplementary Material



## Data Availability

The results of this work were previously disseminated on the arXiv preprint repository: Tao, M.; Larocque, H.; Gyger, S.; Colangelo, M.; Medeiros, O.; Christen, I.; Sattari, H.; Choong, G.; Petremand, Y.; Prieto, I., Yu, Y.; Steinhauer, S.; Leake, G.L.; Coleman, D.J., Ghadimi, A.H.; Fanto, M.L.; Zwiller, V.; Englund, D.; Errando-Herranz, C. Single-photon detectors on arbitrary photonic substrates. 2024, arXiv:2409.08412. arXiv preprint. 10.48550/arXiv.2409.08412 (accessed April 11, 2025).

## Notes

Additional information about device fabrication, characterization, and modeling (PDF)

## References

[1] Ladd T. D., Jelezko F., Laflamme R., Nakamura Y., Monroe C., O’Brien J. L. (2010). Quantum Computers. Nature.

[2] Gisin N., Thew R. (2007). Quantum Communication. Nat. Photonics.

[3] Aspuru-Guzik A., Walther P. (2012). Photonic Quantum Simulators. Nat. Phys..

[4] O’Brien J. L., Furusawa A., Vučković J. (2009). Photonic Quantum Technologies. Nat. Photonics.

[5] Ferrari S., Schuck C., Pernice W. (2018). Waveguide-integrated
superconducting
nanowire single-photon detectors. Nanophotonics.

[6] Reddy D. V., Nerem R. R., Nam S. W., Mirin R. P., Verma V. B. (2020). Superconducting
nanowire single-photon detectors with 98% system detection efficiency
at 1550 nm. Optica.

[7] Colangelo M., Walter A. B., Korzh B. A., Schmidt E., Bumble B., Lita A. E., Beyer A. D., Allmaras J. P., Briggs R. M., Kozorezov A. G., Wollman E. E., Shaw M. D., Berggren K. K. (2022). Large-Area
Superconducting Nanowire Single-Photon Detectors for Operation at
Wavelengths up to 7.4 um. Nano Lett..

[8] Chiles J., Charaev I., Lasenby R., Baryakhtar M., Huang J., Roshko A., Burton G., Colangelo M., Van Tilburg K., Arvanitaki A., Nam S. W., Berggren K. K. (2022). New Constraints
on Dark Photon Dark Matter with Superconducting Nanowire Detectors
in an Optical Haloscope. Phys. Rev. Lett..

[9] Münzberg J., Vetter A., Beutel F., Hartmann W., Ferrari S., Pernice W. H. P., Rockstuhl C. (2018). Superconducting
Nanowire Single-Photon
Detector Implemented in a 2D Photonic Crystal Cavity. Optica.

[10] Gol’tsman G. N., Okunev O., Chulkova G., Lipatov A., Semenov A., Smirnov K., Voronov B., Dzardanov A., Williams C., Sobolewski R. (2001). Picosecond Superconducting Single-Photon
Optical Detector. Appl. Phys. Lett..

[11] Gyger S., Zichi J., Schweickert L., Elshaari A. W., Steinhauer S., Covre da Silva S. F., Rastelli A., Zwiller V., Jöns K. D., Errando-Herranz C. (2021). Reconfigurable Photonics with On-Chip Single-Photon
Detectors. Nat. Commun..

[12] Najafi F., Dane A., Bellei F., Zhao Q., Sunter K. A., McCaughan A. N., Berggren K. K. (2015). Fabrication Process
Yielding Saturated
Nanowire Single-Photon Detectors With 24-ps Jitter. IEEE J. Sel. Top. Quantum Electron..

[13] Sayem A. A., Cheng R., Wang S., Tang H. X. (2020). Lithium-niobate-on-insulator
waveguide-integrated superconducting nanowire single-photon detectors. Appl. Phys. Lett..

[14] Elshaari A.
W., Pernice W., Srinivasan K., Benson O., Zwiller V. (2020). Hybrid Integrated
Quantum Photonic Circuits. Nat. Photonics.

[15] Kim J.-H., Aghaeimeibodi S., Carolan J., Englund D., Waks E. (2020). Hybrid integration
methods for on-chip quantum photonics. Optica.

[16] Najafi F., Mower J., Harris N. C., Bellei F., Dane A., Lee C., Hu X., Kharel P., Marsili F., Assefa S., Berggren K. K., Englund D. (2015). On-Chip Detection of Non-Classical
Light by Scalable Integration of Single-Photon Detectors. Nat. Commun..

[17] Si M., Wang C., Yang C., Peng W., You L., Li Z., Zhang H., Huang J., Xiao Y., Xiong J., Zhang L., Pan Y., Ou X., Wang Z. (2022). Superconducting
NbN thin films on various (X/Y/Z-cut) lithium niobate substrates. Supercond. Sci. Technol..

[18] Lomonte E., Wolff M. A., Beutel F., Ferrari S., Schuck C., Pernice W. H. P., Lenzini F. (2021). Single-photon detection
and cryogenic
reconfigurability in lithium niobate nanophotonic circuits. Nat. Commun..

[19] Colangelo M., Zhu D., Shao L., Holzgrafe J., Batson E. K., Desiatov B., Medeiros O., Yeung M., Loncar M., Berggren K. K. (2024). Molybdenum
Silicide Superconducting Nanowire Single-Photon Detectors on Lithium
Niobate Waveguides. ACS Photonics.

[20] Fahrenkopf N. M., McDonough C., Leake G. L., Su Z., Timurdogan E., Coolbaugh D. D. (2019). The AIM Photonics MPW: A Highly Accessible Cutting
Edge Technology for Rapid Prototyping of Photonic Integrated Circuits. IEEE J. Sel. Top. Quantum Electron..

[21] PsiQuantum
Team (2025). A manufacturable platform
for photonic quantum computing. Nature.

[22] Justice J., Bower C., Meitl M., Mooney M. B., Gubbins M. A., Corbett B. (2012). Wafer-scale integration of group
III–V lasers
on silicon using transfer printing of epitaxial layers. Nat. Photonics.

[23] Larocque H., Buyukkaya M. A., Errando-Herranz C., Papon C., Harper S., Tao M., Carolan J., Lee C.-M., Richardson C. J. K., Leake G. L., Coleman D. J., Fanto M. L., Waks E., Englund D. (2024). Tunable quantum emitters
on large-scale foundry silicon
photonics. Nat. Commun..

[24] Korzh B. (2020). Demonstration of Sub-3 Ps Temporal Resolution with
a Superconducting
Nanowire Single-Photon Detector. Nat. Photonics.

[25] Bandyopadhyay, S. ; Englund, D. Alignment-Free Photonic Interconnects. Preprint at https://arxiv.org/abs/2110.12851, 2021.

[26] Bower, C. A. ; Meitl, M. ; Radauscher, E. ; Bonafede, S. ; Pearson, A. ; Raymond, B. ; Vick, E. ; Verreen, C. ; Weeks, T. ; Gomez, D. ; Moore, T. ; Rotzoll, B. Printing MicroLEDs and MicroICs for Next Generation Displays. https://www.xdisplay.com/wp-content/uploads/2020/05/2018_08_30_IMID-updated.pdf, 2018.

[27] Łysień M., Witczak Ł., Wiatrowska A., Fiaczyk K., Gadzalińska J., Schneider L., Strek W., Karpiński M., Kosior Ł., Granek F., Kowalczewski P. (2022). High-Resolution
Deposition of Conductive and Insulating Materials at Micrometer Scale
on Complex Substrates. Sci. Rep..

[28] Ruf M., Wan N. H., Choi H., Englund D., Hanson R. (2021). Quantum Networks
Based on Color Centers in Diamond. J. Appl.
Phys..

[29] Kok P., Munro W. J., Nemoto K., Ralph T. C., Dowling J. P., Milburn G. J. (2007). Linear optical quantum
computing with photonic qubits. Rev. Mod. Phys..

[30] Slussarenko S., Weston M. M., Chrzanowski H. M., Shalm L. K., Verma V. B., Nam S. W., Pryde G. J. (2017). Unconditional
violation of the shot-noise
limit in photonic quantum metrology. Nat. Photonics.

[31] Reens D., Collins M., Ciampi J., Kharas D., Aull B. F., Donlon K., Bruzewicz C. D., Felton B., Stuart J., Niffenegger R. J., Rich P., Braje D., Ryu K. K., Chiaverini J., McConnell R. (2022). High-Fidelity Ion State Detection
Using Trap-Integrated Avalanche Photodiodes. Phys. Rev. Lett..

