# Supplementary material for: Single-Photon Detectors on Arbitrary Photonic Substrates
Source: ACS Photonics. 2025 Apr 22;12(5):2325–30. doi: 10.1021/acsphotonics.5c00345 (PMC12100732; doi:10.1021/acsphotonics.5c00345)
Supplement: Supplementary file 1 [file ph5c00345_si_001.pdf]

Supporting Information: Single-photon detectors on arbitrary photonic substrates

Max Tao,[†,∇] Hugo Larocque,[†,∇] Samuel Gyger,[†,‡] Marco Colangelo,[†] Owen Medeiros,[†] Ian Christen,[†] Hamed Sattari,[¶] Gregory Choong,[¶] Yves Petremand,[¶] Ivan Prieto,[¶] Yang Yu,[§] Stephan Steinhauer,[‡] Gerald L. Leake,[‖] Daniel J. Coleman,[‖] Amir H. Ghadimi,[¶] Michael L. Fanto,[⊥] Val Zwiller,[‡] Dirk Englund,[†] and Carlos Errando-Herranz[*,†,#,@,△]

†*Research Laboratory of Electronics, Massachusetts Institute of Technology, Cambridge, MA 02139, USA*

‡*KTH Royal Institute of Technology, 106 91 Stockholm, Sweden*

¶*Centre Suisse d'Electronique et de Microtechnique (CSEM), 2000 Neuchâtel, Switzerland*

§*Raith America Inc., Troy, NY 12180, USA*

‖*State University of New York Polytechnic Institute, Albany, NY 12203, USA*

⊥*Air Force Research Laboratory, Information Directorate, Rome, NY 13441, USA*

#*Institute of Physics, University of Münster, Münster 48149, Germany*

@*QuTech and Kavli Institute, Delft University of Technology, 2628 Delft, The Netherlands*

△*Department of Quantum and Computer Engineering, Delft University of Technology, 2628 Delft, The Netherlands*

∇*Contributed equally to this work*

E-mail: c.errandoherranz@tudelft.nl

S1 SNSPD fabrication

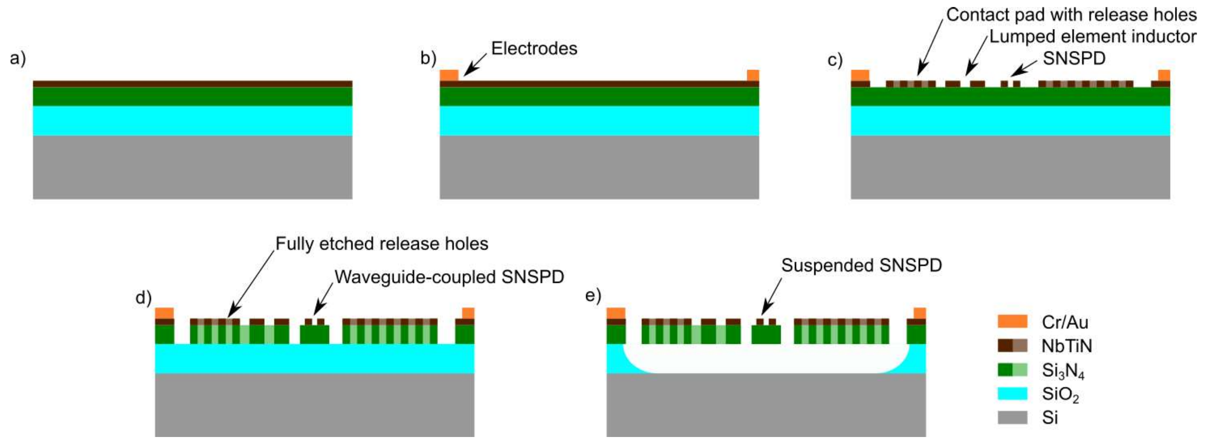

Figure S1: **SNSPD fabrication flow.** Diagrams of the fabricated SNSPDs at various stages of their fabrication flow.

Figure S1 shows the overall fabrication process flow for the SNSPD chiplets. We fabricate the waveguide-coupled SNSPDs from a foundry silicon nitride on insulator wafer with 250 nm of stoichiometric LPCVD silicon nitride and 2 µm of buried oxide. First, we deposit 9 nm of NbTiN on the wafer and then pattern nanostructures using electron beam lithography followed by a directional reactive ion etch. We specifically rely on the fabrication flow presented in,[S1] which deposits the same 9-nm-thin $Nb_{0.86}Ti_{0.14}N$ film via reactive co-sputtering of Nb (200W, DC) and Ti (200W, RF) targets at room temperature in a N and Ar atmosphere. The patterned superconducting film includes a 90 nm wide hairpin nanowire detector connected to a lumped element inductor and contact pads with release holes for under-etching. We optimized the width of the nanowire to both provide sufficient fabrication yields and near-unity internal efficiency. Specifically, wider wires have higher fabrication yields as they are less affected by film defects, yet narrower wires provide higher efficiencies. As for our inductor, its 200 µm length is sufficiently large to avoid latching.[S2] We then pattern the silicon nitride to produce the device's waveguide and release holes. The waveguide width is 1 µm and tapers down to 100 nm over a 10 µm length. Beyond this tapered region, the detector waveguide preserves its nominal 1 µm width over 90 µm. This

length completely overlaps with the extent of the structure's hairpin detector. Tethers connect these waveguides to pads with release holes serving as the main point of contact with the elastomer stamp during transfer printing while providing the required adhesion with the PIC during its placement. These pads occupy an area of 107.5 µ m × 37.5 µm, thereby amounting to a total area 107.5 µm × 79 µm of for the transferred unit. These tethers have a width of 300 nm and a length of 2.1 µm which provides high mechanical stiffness, thereby preventing them from breaking during the transfer process outlined in Section S2. We finally release the resulting structure using a buffered oxide etch followed by critical point drying to remove the buried oxide below the nitride film. Additional tethers with a width of 600 nm and a length of 8 µm connect the structure's transfer pads to the rest of the substrate. These dimensions allow high enough stiffness to keep the structures suspended during the release process, while allowing them to break off during the transfer process outlined in Section S2. NbTiN also covers them to enable post-release electrical screening measurements of the suspended detectors. We implement both sets of tethers within the sample's nominal LPCVD silicon nitride layer. Figure S2 summarizes the aforementioned dimensions for the fabricated detectors.

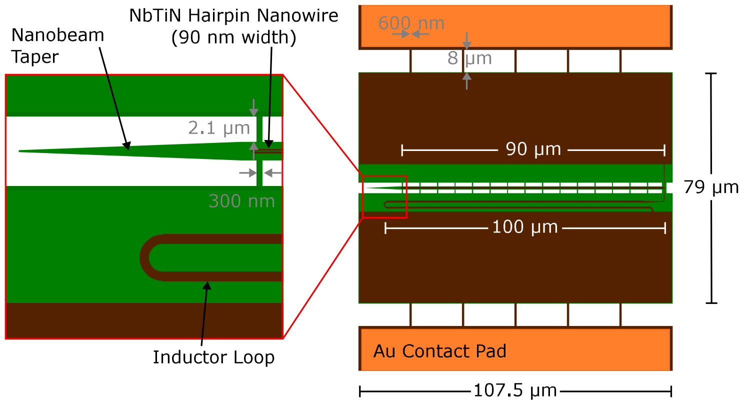

Figure S2: **Feature dimensions of hybrid detectors.** Schematics of the transferred detectors with feature dimensions for its constituent tethers, nanowire, inductor, and transfer pads.

After the full fabrication process, we measured the resistance across the nanowire to

ensure they are still intact and screen for faulty devices showing an open circuit. Figure S3 provides an optical micrograph of a SNSPD sample at the end of the fabrication flow and highlights the components relevant for device screening. Based on nanowire resistivity measurements conducted at room temperature, we observe that 68% (sample size 131) show resistivity between 1 and 2 MΩ at the end of our process. This contrasts the mean 1.2 MΩ (sample size 32, standard deviation 0.3 MΩ, median 1.3 MΩ, interquartile range IQR 0.45 MΩ) measured before HF undercut, and is likely caused by detector damage arising from exposure to HF.

Moreover, we cryogenically pre-characterized 32 devices before BHF release and found a mean switching current of 18 µA (median 19.15 µA, IQR 4.1 µA). In these measurements we observed one outlier of 7.7 µA, that never reached a detection plateau. Figure S4 shows photon and dark count measurements for three representative SNSPDs, flood illuminated at 1550 nm and measured at 2.5 K.

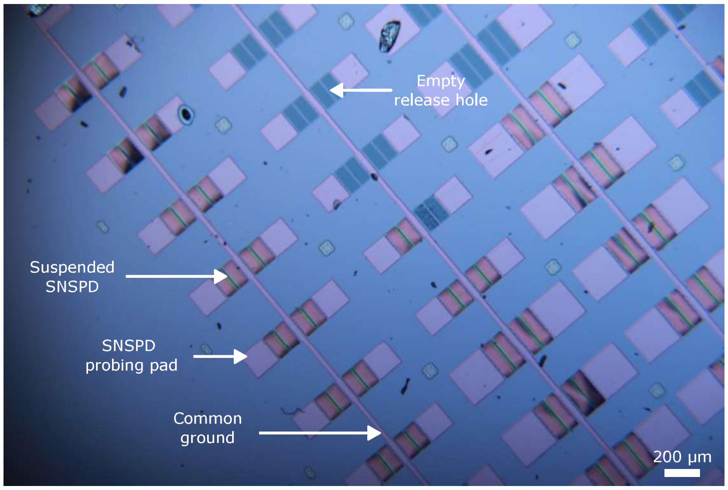

Figure S3: **Fabricated sample with suspended SNSPDs.** Optical micrograph of a fabricated sample with suspended SNSPDs. Connections to probing pads and a common ground allow us to screen individual detectors prior to transferring them to PICs. The image also shows emptied release holes following the successful transfer of a device.

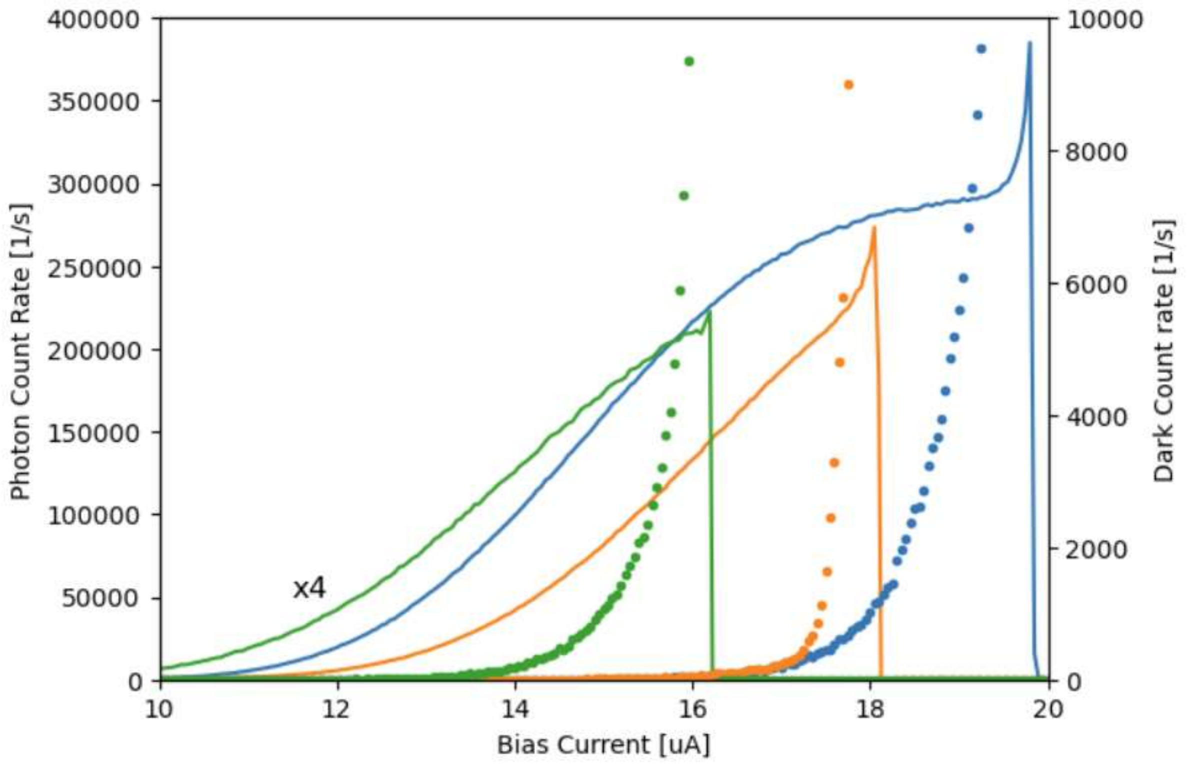

Figure S4: **Representative examples of pre-characterization of SNSPDs before BHF release.** Photon count rate and dark count rates of 3 (out of 32) of our fabricated SNSPDs prior to BHF undercut. The measurements were performed at 2.5 K and with flood illumination at a wavelength of 1550 nm.

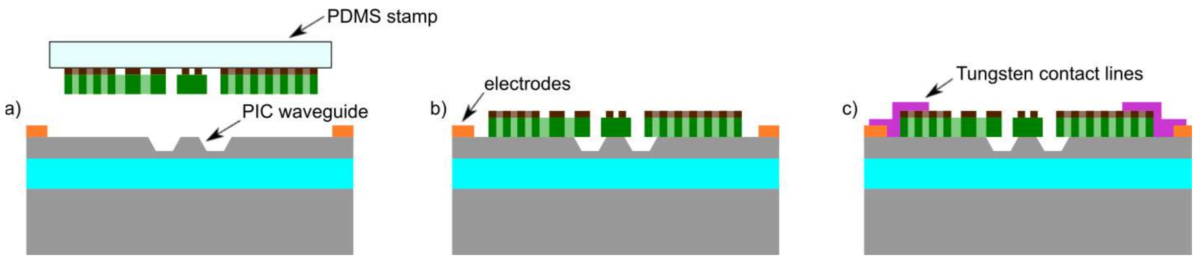

Figure S5: **Hybrid integration fabrication flow** Diagrams of the hybrid SNSPD - PIC structure at various stages of the fabrication flow.

S2 Hybrid structure fabrication

The process flow for the fabrication of the hybrid structure follows Fig. S5. For the case of the commercial silicon PICs, we first pattern gold wirebonding pads around the PIC waveguide to which we can electrically connect the SNSPDs for testing. Specifically, we rely on a post-foundry fabrication scheme involving optical lithography, electron-beam physical vapor deposition of 10 nm chromium and 40 nm of gold, followed by an overnight liftoff process. This process is not necessary for our integration method, and was only used to make the FIB-deposited wiring shorter and thus faster in time. For the LNOI chip we used the pre-existing foundry metals without any post-process metalization step.

Using a 50 µm × 50 µm × 50 µm PDMS stamp, we then transfer the released devices to the photonic chip while optically aligning the waveguide-coupled SNSPD to the corresponding waveguide on the PIC.

To wire our SNSPDs' superconducting film to our chips' contact pads, we deposited tungsten lines using a Raith VELION FIB-SEM system with an Au+ beam set to a 35 kV acceleration voltage, a 120 pA current, and a dose of 2 nC/ µm^2. We set the system to deposit lines that were 1 µm wide and 5 µm long. Our ion beam settings resulted in 500 nm thick lines with residual sputtering from the beam tail up to 2 µm from the targeted deposition sites. For PICs featuring strong insulating features, we found that a specific part of the detector's superconducting hairpin became susceptible to damage during the FIB writing process. We label this region in the diagram shown in Fig. S6(a). Figure S6(b) shows a scanning electron microscope (SEM) image of this region for a functioning device whereas Fig. S6(c) illustrates a detector that has been damaged during FIB deposition. We attribute this damage to localized charging of the transferred detector. The resulting defects exclusively occurred on the silicon PIC and we attribute this phenomenon to its thick silicon dioxide cladding that made it prone to charging. Allowing contact between the detector's transfer pads and the PIC's electronic circuitry prevented this issue, as it provides a discharge line for the detector during the metal deposition process.

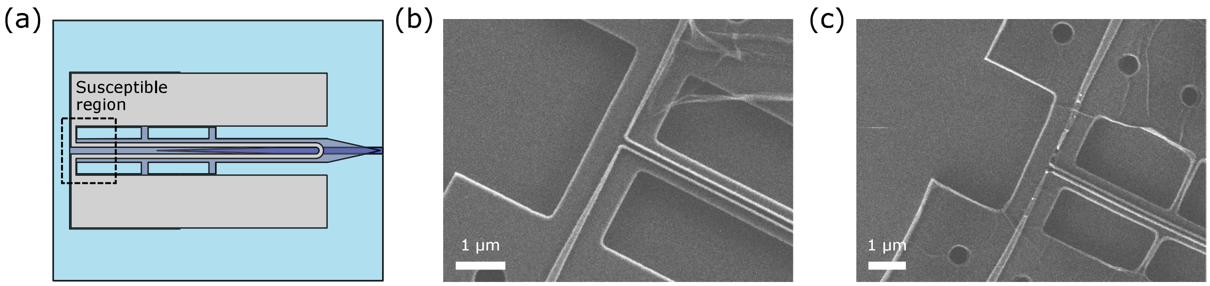

Figure S6: **Detector damage incurred during focused ion beam deposition.** (a) Detector schematics highlighting the part of the superconducting hairpin susceptible to damage. (b) Scanning electron microscope image of the susceptible region for a functioning detector. (c) Scanning electron microscope image of the susceptible region damaged by the focused ion beam.

S3 Experimental setup

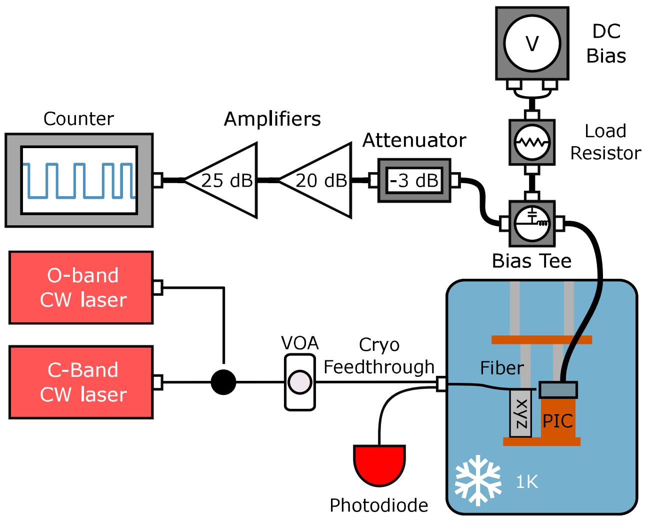

Figure S7: **Apparatus for cryogenic testing.** Figure legend: VOA: Variable Optical Attenuator, PIC: Photonic integrated circuit, xyz: xyz piezo-electrically actuated positioner.

Figure S7 illustrates the schematics for the apparatus used to characterize our hybrid integrated SNSPDs at cryogenic temperatures. For testing the detectors on silicon PICs, we mount our hybrid chips in an ICEoxford 1K cryostat, where a UHNA1 optical fiber array mounted on a 3-axis Attocube piezo positioner stack provides the required optical input/output to the circuit by aligning it to its edge couplers.[S3] Splices to SMF28 fibers

fed through the cryostat ensure optical connectivity to components outside the chamber. These components notably include continuous wave O-band and C-band O-band tunable external cavity diode lasers (Santec TSL-570 and Santec TSL-710), which consisted of the main source of photons for our experiments, along with a variable optical attenuator (JDS HJA9) limiting their output power. While testing the lithium niobate hybrid PICs operating at visible wavelengths, we replaced these tunable sources with a fiber-coupled laser source emitting light near a 650 nm wavelength (Thorlabs S4FC series). We glued the chip to a custom-machined copper plate with thermally-conductive glue and wire bonded it to a printed circuit board. We perform room temperature alignment of the fiber array to the PIC edge couplers first by monitoring optical power on a photodiode (Thorlabs S122C) through a loop-back structure on the chip. A feed-back loop optimizing the strength of this signal then preserves the alignment of the fiber array relative to the PIC while cooling down the chamber. To monitor single photon counts coming from the SNSPDs, we biased them with a DC current using a combination of a bias tee (Mini-Circuits ZFBT-4R2G+), a load resistor (100 kΩ), and voltage source (SRS SIM928). A sequence of components consisting of an attenuator and low-noise amplifiers (RF Bay LNA-2500, LNA-2000) amplified the acquired biased signal before sending it to a counter (Agilent 53131a).

S4 Detector efficiency modeling

Low overlap between the hybrid mode and the SNSPD over most of the chiplet's length could be responsible for the low optical detection efficiencies (ODE) in the hybrid silicon PICs. Here, the PIC waveguides specifically consists of 220 nm-thick and 400 nm-wide silicon waveguides designed for single-mode O- and C+L-band operation. Underneath the transferred silicon nitride waveguides, their widths taper down to 200 nm over a length of 40 µm. As for the detector waveguide, it consists of a 250 nm-thick and 1 µm-wide silicon nitride. It maintains a uniform cross-section underneath the hairpin detector, which extends

over a length of 90 µm. Outside this region, the taper involved in the hybrid mode conversion is 10 µm long and has a width that linearly goes down from 1 µm to 100 nm. A 100 nm-thick layer of silicon dioxide separates the detector and PIC waveguides. This thickness is a fixed parameter set by the foundry that manufactured the silicon PIC. As addressed in,[S4] adjusting this thickness can potentially lead to better mode conversion from the PIC to detector waveguide. Such optimization usually involves picking a thickness large enough to ensure adiabatic evolution and minimize reflections at the tip of the detector taper. At the same time, we must keep it small enough to ensure sufficient coupling between the two waveguides. As shown in Fig. 1(c) of the main text, the tip of the silicon nitride waveguide minimally perturbs the mode structure of the silicon waveguide, thus enabling a smooth transition in the mode structure of the underlying mode converter. Therefore, adopting a thinner thickness would likely increase the coupling strength between the two waveguides while not significantly disrupting the evolution of the propagating mode, thereby yielding potentially higher detection efficiencies.

When the constituent waveguides of the detector's hybrid mode converter are perfectly aligned, we expect the converter's fundamental mode to mostly contain light coming from the PIC's waveguide. We expect the evolution of this mode, $A(x, y)$, to verify

$$\frac{\partial A}{\partial z} = (-ik(z) + \alpha(z))A(z), \tag{S1}$$

$$A(z, \Delta z) \propto \exp\left(\int_{z'=z}^{z'=z+\Delta z} -ik(z') + \alpha(z')\, dz'\right), \tag{S2}$$

where $A(z)$ is the weight of the mode as it propagates along z, while $k(z)$ and $\alpha(z)$ are the z-dependent real and imaginary components of the mode's propagation constants. The ODE of the device thus becomes $1 - |A(L)|^2$, where L is the length of the hybrid structure. Losses incurred at abrupt changes in the hybrid mode converter can further alter this metric. We expect these losses to primarily arise at the tip of the mode converter's tapers. Assuming that losses at the tip of the detector and PIC waveguide tapers are given by t_{det} and t_{PIC},

respectively, then the expected ODE of our hybrid detectors corresponds to

$$|t_{\text{det}}|^2(1-|A(z_1,\Delta z_1)|^2)+|t_{\text{det}}|^2|t_{\text{PIC}}|^2|A(z_1,\Delta z_1)|^2(1-|A(z_2,\Delta z_2)|^2) \tag{S3}$$

where we schematically define the positions $z_{1,2}$ and intervals $\Delta z_{1,2}$ in Fig. S8. From finite

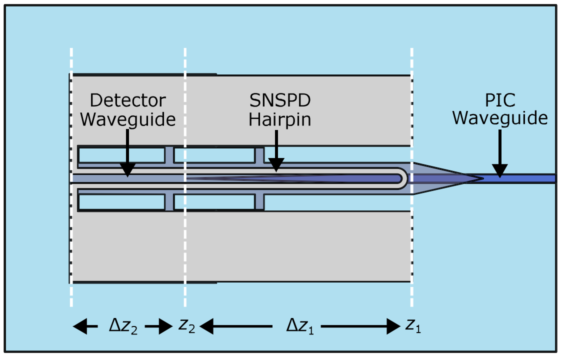

Figure S8: **Parameters related to the hybrid detector efficiency**. z_1 and z_2 correspond to the start of the hairpin diode and to the end of the PIC waveguide, respectively. We separate the length of the hairpin among $\Delta z_1 = z_2 - z_1$ and Δz_2.

difference time domain (FDTD) simulations, we estimate $|t_{\text{det}}|^2 = 0.995$ and $|t_{\text{PIC}}|^2 = 0.925$. To evaluate the $A(z, \Delta z)$ terms, we rely on numerically calculated values of the effective refractive indices of the fundamental mode along the mode converter using a finite element method (FEM). From these indices, we extract the propagation constants $k(z)$ and $\alpha(z)$, thus allowing us to compute $A(z, \Delta z)$ using Eq. (S2). From these simulations, Eq. (S3) suggests an expected detection efficiency of 30.3% for a perfectly transferred SNSPD on our silicon PIC operating near a wavelength of 1570 nm.

When the transferred detector exhibits a rotation offset from its perfectly aligned configuration as sketched out in Fig. S9(a), the resulting asymmetries lead to the excitation of higher order modes in the hybrid mode converter. Such alignment deviations are inherent to the limited resolution provided by the transfer printing apparatus' imaging system. Prior work relying on the same system to transfer quantum emitters on foundry silicon photonics suggests alignment precisions of 202 nm and 0.59° for the lateral and rotational offset of the transferred SNSPD with respect to the underlying PIC waveguide, respectively.[S4] We de-

fine these two offsets in Fig. S9(a). The resulting coupling between the device's eigenmodes therefore causes Eqs. (S2,S3) to lose their validity. To capture such effects, we numerically propagate the fundamental TE mode of the silicon PIC waveguide through the mode converter using an FEM method implemented in RSoft Photonic Device Tools. The software provides the energy absorbed by the device, which it calculates using the following integral,

$$U_A = \omega \int_V \mathrm{Im}[\epsilon(\omega)] |\mathbf{E}(\omega)|^2 \, dV \tag{S4}$$

where ω is the optical frequency, ϵ is the dielectric permittivity and $\mathbf{E}$ is the electric field. Our model assumes that only the NbTiN hairpin has a non-zero imaginary permittivity. Specifically, we consider real and imaginary refractive indices of 4.2071 and 3.8736, respectively, for this superconductor.[S5] For a straight waveguide, U_A is proportional to the $1 - |A(L)|^2$ expression used to calculate efficiency. To estimate the influence of misalignment on our ODE, we therefore normalize these absorbed energy values to the 30.3% ODE expected from the effective indices of the TE0 mode for a perfectly aligned device. Figure S9(b) provides these normalized efficiency values for hybrid structures with detector waveguides exhibiting various rotational offsets with respect to the PIC waveguide. Optical imaging of the hy-

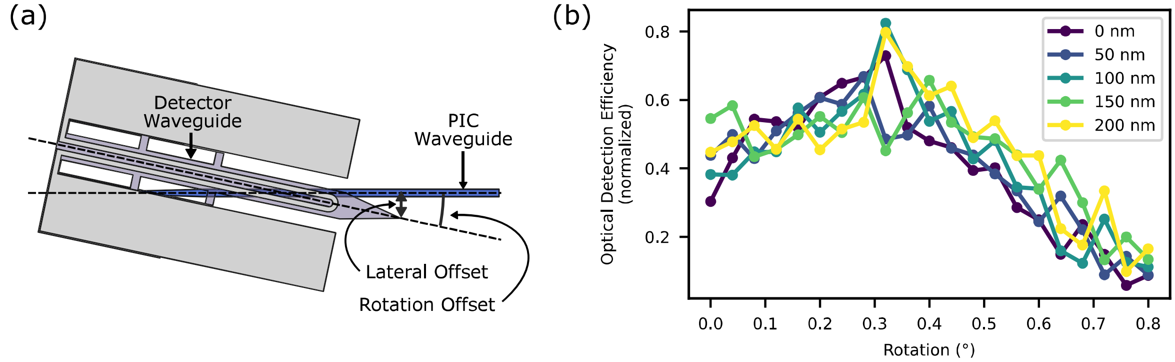

Figure S9: **Expected optical detection efficiency for hybrid detectors**. (a) Diagram illustrating the observed rotational offset incurred during the transfer of our detectors on silicon PICs. (b) We consider detectors transferred on a silicon PIC operating at an optical wavelength of 1570 nm and featuring various lateral and rotational offsets between their constituent waveguides.

brid device considered in this work indicates a 0.8° offset between the two waveguides. We specifically rely on an objective with a NA of 0.95 for this imaging task. We extract this value by visually locating two points along each waveguide, one of which lying on the tip of the detector. The lines connecting each point allow us to deduce the rotational offset between the two waveguides. The distance between the tip of the detector from the line attributed to the PIC waveguide then gives us its lateral offset, which we then convert from pixel units to physical units with a known 10 µm feature in another image taken under the same conditions.[S4] We illustrate this extraction procedure in Fig. S10. From Fig. S9(b), we expect this offset to reduce our ODE near 8.7%, which is near our $7.8 \pm 0.2\%$ measured value. We attribute the increased ODE at intermediate rotation angles to the excitation of the hybrid structure's TM0 mode,[S6] which features greater overlap with the device's SNSPD, thereby leading to enhanced optical absorption. Compared to the TE0 mode's 30.3% ODE for a perfectly aligned mode converter, FEM simulations suggest that sending the silicon waveguide's TM0 mode into the detector would result in absorption attributed to a 97.4% ODE. However, we are not able to verify this figure directly in our experiment. Specifically, given our circuit's configuration, substantial and spectrally varying TM insertion losses in our silicon PIC's various components prevent us from reliably calibrating out these contributions from the counts monitored on the SNSPD. Figure S9(b) also shows the expected ODE for various lateral offsets of the detector waveguide as defined in Fig. S9(a). Within the expected alignment precision of our transfer printing apparatus, we observe minimal variations with this lateral offset in comparison to those in the rotational offset. We attribute these small variations to the long length over which the detector waveguide couples to the PIC waveguide. Fluctuations in the data likely arise from the finite resolution of our simulation set to run over a reasonable duration. In particular, we consider a 6 µm × 2.3 µm simulation window extending over a 105 µm length with a 20 nm × 20 nm resolution.

Co-designing the PIC and detector waveguides in the hybrid device would increase its detection efficiency. Given the constraint of working with the considered 250 nm × 1 µm

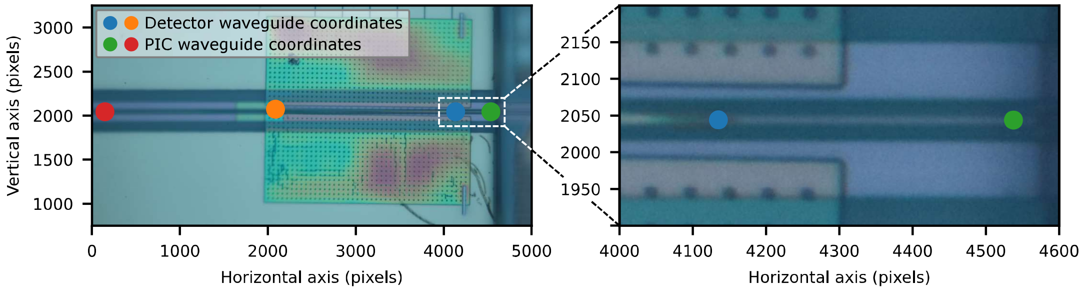

Figure S10: **Alignment extraction.** Image of the transferred SNSPD on the silicon PIC along with the coordinates for extracting the relative offset between the detector and PIC waveguides on the silicon hybrid circuit. Inset: Image section focusing on the tip of the detector waveguide.

silicon nitride detector waveguides integrated on a silicon photonic PIC, such co-design could involve using a silicon nitride PIC waveguide, which are increasingly prevalent in commercial silicon PIC manufacturing processes. For the specific case of the AIM Photonics foundry process used in this work, silicon PICs can be manufactured with an additional layer of silicon nitride waveguides over the PIC's principal layer of silicon waveguides. Process design kit components known as 'escalators' can efficiently route optical signals between the two layers with insertion losses of less than 0.1 dB.[S7] Therefore, integrating detectors onto foundry silicon PICs could leverage this additional layer to provide better detection efficiencies. To illustrate, at the considered 1570 nm optical wavelength, routing light from the PIC's silicon waveguide into a 220 nm thick silicon nitride waveguide that tapers from a width of 1 µm down to 200 nm over a length of 20 µm can increase the ODE near 80 %. At this point, the overlap between the detector waveguide mode and the SNSPD primarily limits detection efficiency. Besides increasing this overlap with a modified detector waveguide geometry, increasing the length of this waveguide provides another venue for increasing the ODE. For example, we estimate that a detector waveguide length of 250 µm ought to increase efficiency above 99%. Such longer detectors would be compatible with transfer printing, as this integration method has been shown to accommodate longer photonic structures exceeding

2 mm.[S8] Alternatively, replacing the silicon nitride detector waveguide by a material with a refractive index closer to the PIC's silicon waveguide could provide better coupling between the two and thus higher detection efficiencies. Such materials could for instance consist of silicon, indium phosphide, or other high-index III-V materials that can be mechanically released.

Table S1 summarizes the efficiency figures reported in this section and in the main text for detectors transferred on silicon and also on lithium niobate PICs. Herein, we provide the maximum measured efficiencies in our experiments, the modeled detection efficiency assuming perfect alignment during the transfer printing part of the fabrication process, and theoretically achievable detection efficiencies for revised PIC and detector waveguide geometries in the two considered platforms.

Table S1: **Obtained and potential detection efficiencies of hybrid integrated SNSPDs on silicon and lithium niobate PICs**. The reported efficiencies include the ones measured in our experiments, the modeled values given our current PIC and detector waveguide geometries assuming perfect alignment between the two constituents, and modeled values that assume design modifications in the PIC and SNSPDs.

PIC Platform	Silicon	Lithium Niobate
Wavelength	1570 nm	650 nm
Measured efficiency	7.6%	8.6%
Modeled efficiency assuming perfect transfer alignment	30.3%	90%
Modeled efficiency with co-designed PICs and SNSPDs	>99%	>99%

S5 Transfer printed SNSPD characterization

Figure S11 shows the electrical response of our hybrid integrated SNSPDs to various bias currents and to a detected single photon. Figure S11(a) plots the IV curve of the device, thereby indicating a switching current of 7.1 µA. Figure S11(b) plots the pulse profile of the detector attributed to the detection of a photon, which features a decay time of $\sim$ 50 ns along with secondary peaks likely attributed to reflections from the amplifiers shown in Fig. S7.

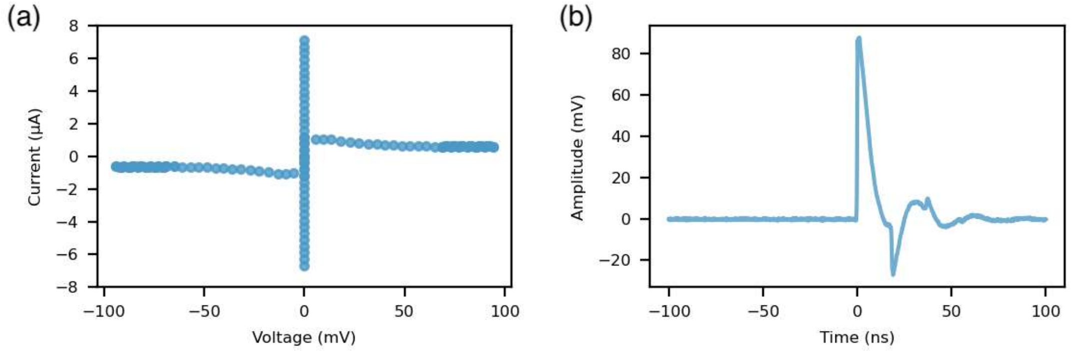

Figure S11: **Electrical response of hybrid integrated SNSPDs at cryogenic temperatures of 1 K.** (a) IV curve of the SNSPD. (b) Pulse profile of the SNSPD following the detection of a photon.

To verify the linear response of our detector's counts to the number of incident photons, we monitor this quantity while decreasing our input's optical power with our variable optical attenuator. Figure S12 plots the results of this measurement and features a distinct linear drop in count-rate as we increase the attenuation of our input. Finally, as shown in Fig. S13, we measure a 242 ps jitter for our integrated detectors. This value is in line with those for devices reported in prior work relying on the same fabrication process and nanowire geometry.[S9] Varying levels of NbTiN film quality across the wafer likely account for such jitter variations. Future work addressing these variations would need to examine the effects of individual steps of our fabrication process on the film quality of the superconductor, specifically those related to the buffered hydrofluoric acid release step.

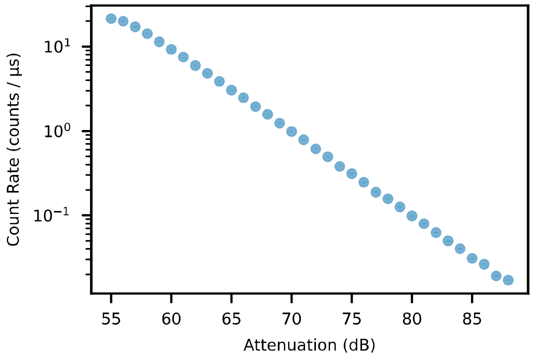

Figure S12: **SNSPD count-rate dependence on incident photon flux.** SNSPD count-rate vs the attenuation of our input optical signal.

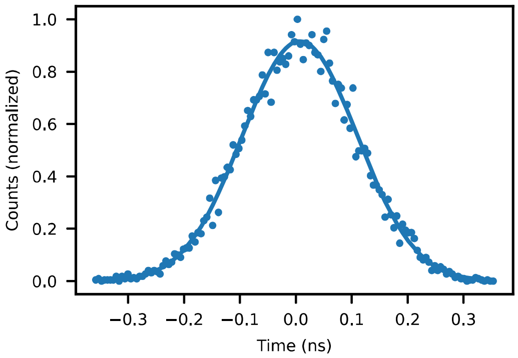

Figure S13: **Jitter measurement.** System jitter measurement indicating a detector jitter of 242 ps.

S6 Off-chip optical transmission calibration

Besides the misalignment of the transferred detectors, insertion losses attributed to the PIC and other fiber optic components cause a drop in incident power with respect to our laser's output. To estimate the optical power incident on our hybrid integrated detectors, we calibrated off-chip transmission losses through the various optical components inserted between our lasers and our PIC's waveguides.

We summarize these values for the apparatus used to characterize our silicon PICs in Table S2. We measured losses due to the cryo feedthrough, i.e. the "cryo feedthrough to fiber facet" value, by measuring optical power coming out of its fiber array while sending light through the spliced input SMF28 fibers. As emphasized in Fig. S14, the value provided in Table S2 corresponds to losses for the fiber coupled to the waveguide leading to the characterized SNSPD. Furthermore, we estimate the average PIC facet loss by measuring transmission through our chip's loopback structure as shown in Fig. S14, removing the contribution of the feedthrough losses of the employed fibers, and assuming that the losses at the two PIC facets equally contribute to the resulting metric. We rely on a single loopback structure, i.e., a sample size of one, to estimate our facet losses. Though measurements attributed to multiple structures would have been beneficial towards estimating an error for

this figure, we expect this error to be low given the consistency of foundry-level manufacturing methods that produced our silicon PIC.[S3,S7] In addition to losses due to components before the feedthrough, the feedthrough itself, and the PIC facet, we further weaken our input signal with the variable optical attenuator shown in Fig. S7. As indicated in Table S2, we set its attenuation to 70 dB while characterizing our SNSPD.

Table S2: **Optical transmission losses of the fiber optics components placed before the PIC.** Transmission losses of the optical components placed between the hybrid silicon PIC and the laser used to characterize them.

Optical Path Stage	1570 nm	1312 nm
Laser to cryo feedthrough	4.18 dB	4.65 dB
Cryo feedthrough to fiber facet	1.35 dB	1.35 dB
Average PIC facet loss	8.35 dB	11.05 dB
Attenuator setting during SNSPD testing	70 dB	70 dB

We assume that the losses listed in Table S2 completely account for the attenuation of the incident optical signal at the hybrid integrated detectors. As illustrated in Fig. S14, unaccounted additional losses could arise from a directional coupler placed between the facet and the SNSPD. Its presence would have allowed simultaneous testing of two detectors with a single optical input. However, wiring issues incurred during the sample's cool-down prevented us from testing the hybrid chip's second detector. To mitigate these losses, we varied the optical wavelength of our input light until the counts on our SNSPD were maximized, thereby suggesting that the coupler routed most of the input light into its bar port. As indicated in Table S2 and in the main text, these optimal wavelength consist of 1570 nm for the C+L band and 1312 nm for the O-band. Since fabrication imperfections typically only limit maximum transmission in the cross port,[S10] any drop in counts due to the directional coupler likely stem from insertion loss. Rigorously accounting for this metric and the corresponding error would require dedicated test structures, which were not included on the PIC due to space constraints.

For the LNOI experiment, the optical losses per set of facet and feedthrough based on transmission measurements through two feedthroughs and edge couplers pairs separated by

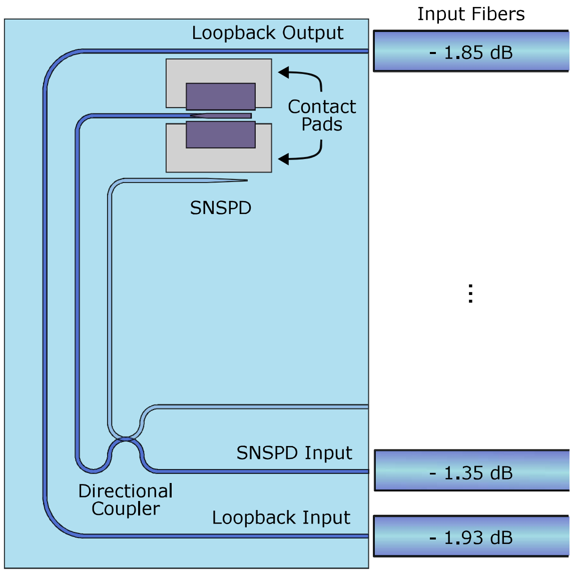

Figure S14: **Schematic of silicon hybrid PIC.** Our testing apparatus relies on three input fibers going through the cryo feedthrough and exhibiting different insertion losses. Two of them couple to an on-chip loopback structure allowing us to calibrate facet losses from the PIC. A directional coupler lies between the last facet and the SNSPD. To minimize its contribution to the measurement, we alter the optical wavelength used in our experiments such that most of the input light gets routed to the coupler's bar port. The schematics also provides the insertion loss of each fiber due to the splices to the cryo feedthrough.

an on-chip splitter were −19.68 dB, −20.09 dB, and −19.73 dB for detectors D1, D2, and D3 respectively. Figure S15 provides an annotated optical micrograph of the circuit used for this measurement. The resulting average transmission through a set of facet and feedthrough is therefore $1.04 \pm 0.05\%$, which we propagate into the uncertainty values reported in the main text. The on-chip splitter was measured to split light by 3 dB at 650 nm. The attenuation was set to 58.9 dB.

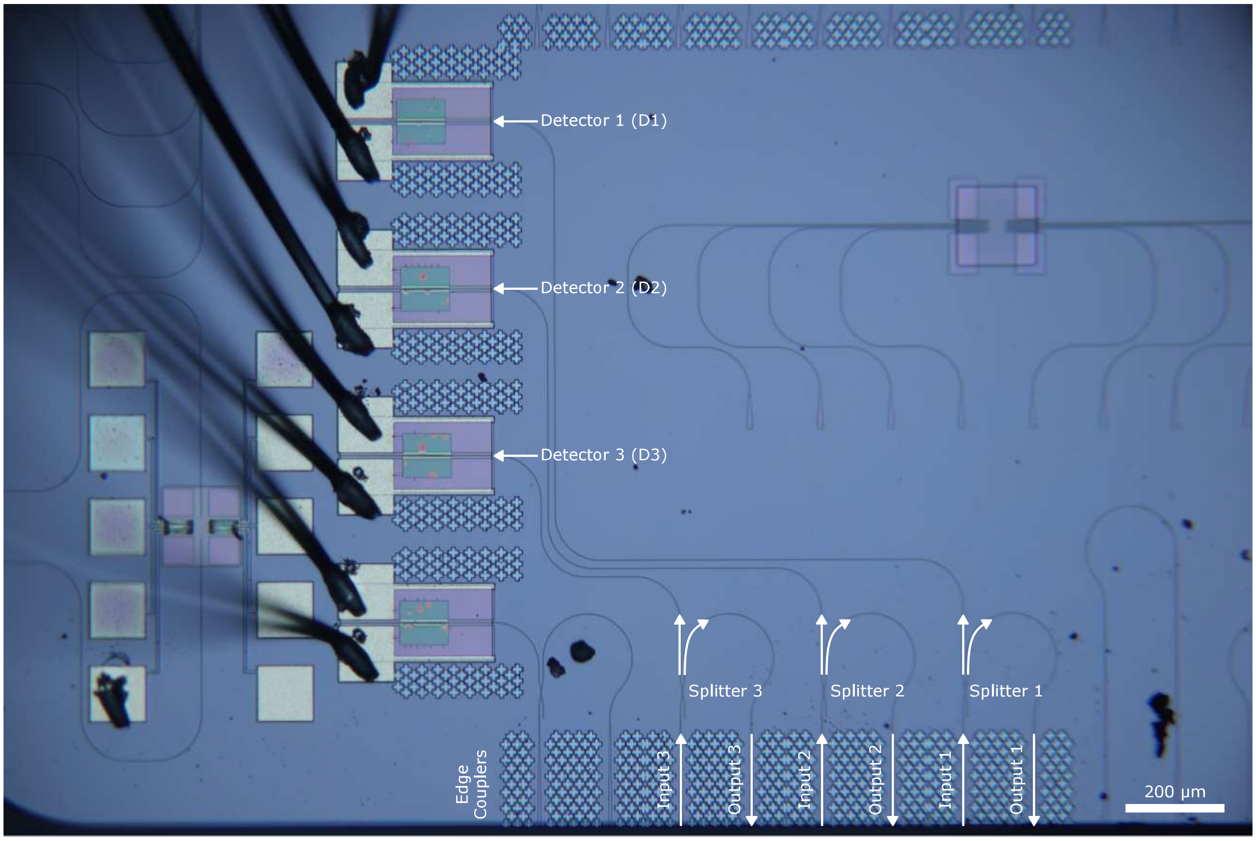

Figure S15: **Optical micrograph of a lithium niobate PIC with three integrated SNSPDs.** Edge couplers channel light from an optical fiber to an on-chip 3 dB splitter. Each slitter routes a portion of the optical signal to the hybrid integrated detectors and the other back to an edge coupler for transmission calibration.

S7 On-chip detection efficiency error

We calculate the error on our reported ODE values based on the error on our measured counts and that of the optical transmission through the fiber array feed-through coupling

light into the PIC, dB_f. Because we extracted the value by manually holding the fiber array in front of a power meter, we expect the resulting fluctuations to dominate the error on the expected photon flux hitting the detector, as defined in Eq. (1) of the main text. We extract the error on the measured counts from the standard deviation of four counts vs bias current traces for input wavelengths of 1570 nm and 1312 nm, as shown in Fig. S16(a,b), respectively. Each data set features slight differences in fiber-to-PIC coupling efficiency. Therefore, we calculate our error based on versions of the traces scaled to the one featuring optimal counts, which correspond to the ones shown in Fig. 2 of the main text. We then use standard propagation of uncertainty methods to extract the error on our ODE defined as $\Phi_{\text{measured}}/\Phi_{\text{expected}}$.

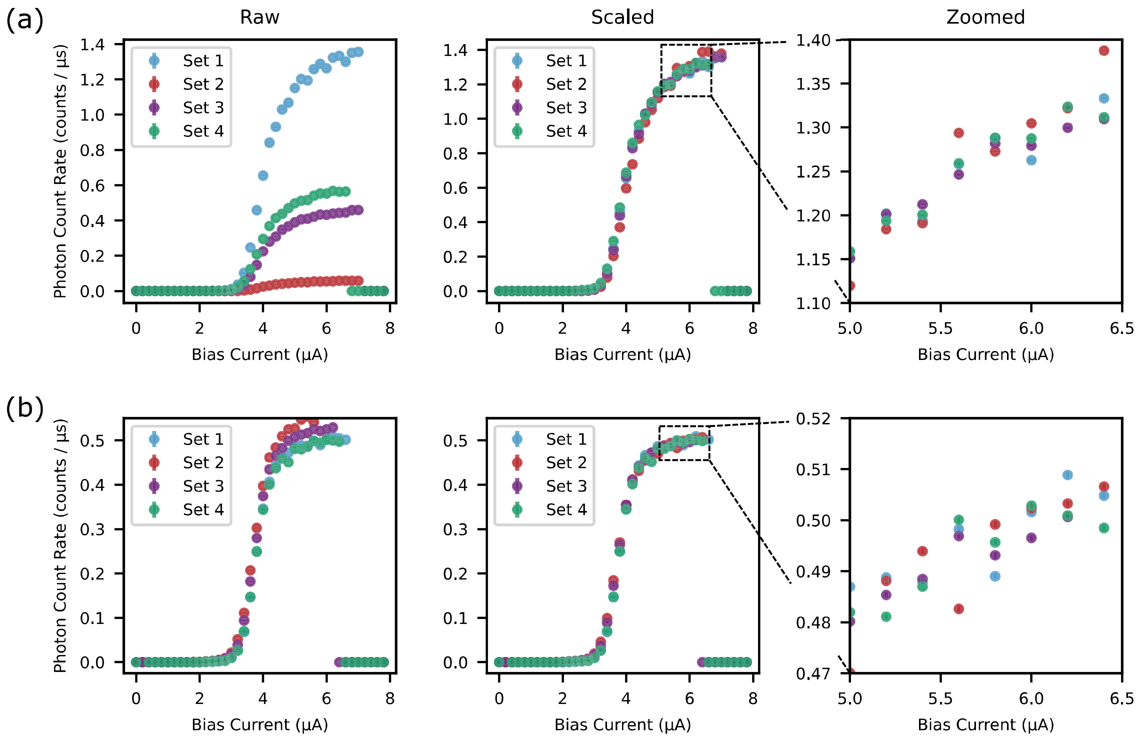

Figure S16: **Sets of photon count traces.** Data used to estimate our the optical detection efficiency of the detector integrated on our silicon PIC. Given varying degrees of fiber-to-PIC coupling among data sets, we estimate the error and the average of our photon count rates based on versions of the traces scaled to the one featuring optimal counts. These optimal traces correspond to the ones shown in Fig. 2 of the main text.

The relevance of this error calculation assumes a low insertion loss variance for the PIC

components placed between the hybrid detectors and the optical fiber sending light into the PIC. It also ignores the potential contributions of stray light and the influence of misalignment between the hybrid SNSPD's PIC and detector waveguides. The method's validity therefore only applies to devices involving consistent PIC components, large separations between the input fiber and detectors, and that consider the influence of transfer misalignment through microscopy and modeling. Though the hybrid detectors transferred on the silicon PIC meet these criteria, the ones on the lithium niobate PIC do not. This drawback explains why the variations in the three reported lithium niobate device efficiencies do not fall within their uncertainties.

S8 Challenges of monolithic integration of SNSPDs on PICs

Our hybrid method aims to provide a single solution to SNSPD integration on PICs regardless of the underlying chips's material platform, size, pattern density, and surface topography. Numerous methods allow for direct monolithic integration of SNSPDs across very targeted photonic platforms.[S11] Typically, these demonstrations showcase the integration of nanowires placed on waveguides using either a top-down (detector-first) or bottom-up (waveguide-first) approach. Excluding process development, the primary challenges associated with integration include incompatibilities between superconducting layer deposition and optical materials, as well as incompatibilities between the etching processes patterning nanowire detectors and photonic waveguides. Consequently, these incompatibilities often lead to increased optical losses due to detector integration or degraded detector performance resulting from waveguide etching. Such complications generally contribute to lower yields observed in integrated systems. The incompatibility of superconducting material deposition with optical platforms typically arises from the requirement for deposition at high temperature to achieve superior superconducting properties. This tends to degrade the optical prop-

erties of the substrate. To address this issue, room-temperature deposition processes have been developed for both polycrystalline and amorphous superconducting thin films.[S12–S15] As for integration issues stemming from etching processes, mitigation measures include amorphous passivation layers (e.g., atomic-layer-deposited hafnium oxide[S13]) deposited between the waveguides and detectors. Other methods rely on protecting nanowires with a thick mask and the development of less aggressive processes to pattern waveguides.[S14]

However, this discussion overlooks a crucial application in which direct integration methods typically fail: integrating detectors onto densely patterned photonic foundry chips. In these chips, detectors are integrated as the final step after the photonic chips have already been fabricated and delivered, thereby significantly increasing integration challenges. Typical issues include difficulties in performing lithography due to significant layer topography, constraints related to chip size, the necessity of pre-processing the chips (such as exposing waveguides by cladding removal), discontinuities in material deposition, and complexities related to DC and RF routing in dense circuits. Incorporating detector fabrication processes during initial foundry production could mitigate these issues.[S16] However, given the need for specialized fabrication tools and the expertise to operate them, such measures are not easily available for prototyping tasks and small to medium volume production.

These challenges highlight the main benefit of our hybrid integration approach: it is entirely agnostic to a PIC's existing features. This advantage makes it broadly applicable compared to direct integration techniques, thereby making SNSPD-integration available to the wider photonics community. In particular, integration onto PICs with a well-established fabrication process becomes immediately accessible without additional process-development encountered in direct integration approaches.[S13–S15] Such accessibility considerably simplifies the development of potentially more sophisticated on-chip systems than what can be achieved with direct deposition on silicon[S17] or lithium niobate platforms[S18,S19] with more customized fabrication. Directly comparing our method with such works would be preliminary given its early development stage. However, future work improving detection efficiencies as outlined

in Section S4 promises competitive performance metrics for hybrid devices.

References

(S1) Zichi, J.; Chang, J.; Steinhauer, S.; von Fieandt, K.; Los, J. W. N.; Visser, G.; Kalhor, N.; Lettner, T.; Elshaari, A. W.; Zadeh, I. E.; Zwiller, V. Optimizing the stoichiometry of ultrathin NbTiN films for high-performance superconducting nanowire single-photon detectors. *Opt. Express* **2019**, *27*, 26579–26587.

(S2) Annunziata, A. J.; Quaranta, O.; Santavicca, D. F.; Casaburi, A.; Frunzio, L.; Ejrnaes, M.; Rooks, M. J.; Cristiano, R.; Pagano, S.; Frydman, A.; Prober, D. E. Reset dynamics and latching in niobium superconducting nanowire single-photon detectors. *J. Appl. Phys.* **2010**, *108*, 084507.

(S3) Timurdogan, E.; Su, Z.; Shiue, R.-J.; Poulton, C. V.; Byrd, M. J.; Xin, S.; Watts, M. R. APSUNY Process Design Kit (PDKv3.0): O, C and L Band Silicon Photonics Component Libraries on 300mm Wafers. Optical Fiber Communication Conference (OFC) 2019. 2019; p Tu2A.1.

(S4) Larocque, H.; Buyukkaya, M. A.; Errando-Herranz, C.; Papon, C.; Harper, S.; Tao, M.; Carolan, J.; Lee, C.-M.; Richardson, C. J. K.; Leake, G. L.; Coleman, D. J.; Fanto, M. L.; Waks, E.; Englund, D. Tunable quantum emitters on large-scale foundry silicon photonics. *Nat. Commun.* **2024**, *15*, 5781.

(S5) Banerjee, A.; Heath, R. M.; Morozov, D.; Hemakumara, D.; Nasti, U.; Thayne, I.; Hadfield, R. H. Optical properties of refractory metal based thin films. *Opt. Mater. Express* **2018**, *8*, 2072–2088.

(S6) Watts, M. R.; Haus, H. A. Integrated mode-evolution-based polarization rotators. *Opt. Lett.* **2005**, *30*, 138–140.

(S7) Fahrenkopf, N. M.; McDonough, C.; Leake, G. L.; Su, Z.; Timurdogan, E.; Coolbaugh, D. D. The AIM Photonics MPW: A Highly Accessible Cutting Edge Technology for Rapid Prototyping of Photonic Integrated Circuits. *IEEE J. Sel. Top. Quantum Electron.* **2019**, *25*, 1–6.

(S8) Vandekerckhove, T.; Vanackere, T.; Witte, J. D.; Cuyvers, S.; Reis, L.; Billet, M.; Roelkens, G.; Clemmen, S.; Kuyken, B. Reliable micro-transfer printing method for heterogeneous integration of lithium niobate and semiconductor thin films. *Opt. Mater. Express* **2023**, *13*, 1984–1993.

(S9) Gyger, S.; Zichi, J.; Schweickert, L.; Elshaari, A. W.; Steinhauer, S.; Covre da Silva, S. F.; Rastelli, A.; Zwiller, V.; Jöns, K. D.; Errando-Herranz, C. Reconfigurable Photonics with On-Chip Single-Photon Detectors. *Nat. Commun.* **2021**, *12*, 1408.

(S10) Chrostowski, L.; Hochberg, M. *Silicon Photonics Design: From Devices to Systems*; Cambridge University Press, 2015; p 92–161.

(S11) Ferrari, S.; Schuck, C.; Pernice, W. Waveguide-integrated superconducting nanowire single-photon detectors. *Nanophotonics* **2018**, *7*, 1725–1758.

(S12) Dane, A. E.; McCaughan, A. N.; Zhu, D.; Zhao, Q.; Kim, C.-S.; Calandri, N.; Agarwal, A.; Bellei, F.; Berggren, K. K. Bias sputtered NbN and superconducting nanowire devices. *Appl. Phys. Lett.* **2017**, *111*, 122601.

(S13) Sayem, A. A.; Cheng, R.; Wang, S.; Tang, H. X. Lithium-niobate-on-insulator waveguide-integrated superconducting nanowire single-photon detectors. *Appl. Phys. Lett.* **2020**, *116*, 151102.

(S14) Lomonte, E.; Wolff, M. A.; Beutel, F.; Ferrari, S.; Schuck, C.; Pernice, W. H. P.; Lenzini, F. Single-photon detection and cryogenic reconfigurability in lithium niobate nanophotonic circuits. *Nat. Commun.* **2021**, *12*, 6847.

(S15) Colangelo, M.; Zhu, D.; Shao, L.; Holzgrafe, J.; Batson, E. K.; Desiatov, B.; Medeiros, O.; Yeung, M.; Loncar, M.; Berggren, K. K. Molybdenum Silicide Superconducting Nanowire Single-Photon Detectors on Lithium Niobate Waveguides. *ACS Photonics* **2024**, *11*, 356–361.

(S16) PsiQuantum Team A manufacturable platform for photonic quantum computing. *Nature* **2025**, DOI: https://doi.org/10.1038/s41586-025-08820-7.

(S17) Waki, K.; Yamashita, T.; Inoue, S.-i.; Miki, S.; Terai, H.; Ikuta, R.; Yamamoto, T.; Imoto, N. Fabrication and Characterization of Superconducting Nanowire Single-Photon Detectors on Si Waveguide. *IEEE Trans. Appl. Supercond.* **2015**, *25*, 1–4.

(S18) Zichi, J.; Gyger, S.; Baghban, M. A.; Elshaari, A. W.; Gallo, K.; Zwiller, V. An NbTiN superconducting single photon detector implemented on a LiNbO3 nano-waveguide at telecom wavelength. Proceedings of the European Conference on Integrated Optics. 2019.

(S19) Prencipe, A.; Gyger, S.; Baghban, M. A.; Zichi, J.; Zeuner, K. D.; Lettner, T.; Schweickert, L.; Steinhauer, S.; Elshaari, A. W.; Gallo, K.; Zwiller, V. Wavelength-Sensitive Superconducting Single-Photon Detectors on Thin Film Lithium Niobate Waveguides. *Nano Lett.* **2023**, *23*, 9748–9752, Publisher: American Chemical Society.
